# What Evidence-based Medicine (EBM) Doesn’t Say About Allergen-specific Immunotherapy (AIT)

**DOI:** 10.37825/2239-9747.1040

**Published:** 2023-09-22

**Authors:** Gabriele Di Lorenzo, Marcello Melluso, Alessandro Rodolico

**Affiliations:** a Department of Health Promotion Sciences, Maternal and Infant Care, Internal Medicine and Medical Specialties (PROMISE), University of Palermo, Palermo, Italy; b Medico di Medicina Generale ASP 206 of Palermo, Palermo, Italy; c Psychiatry Unit, Department of Clinical and Experimental Medicine, University of Catania, 95123 Catania, Italy

**Keywords:** Conflict of interest, Evidence-based medicine, Allergen-specific immunotherapy, Randomized controlled trials, Meta-analysis, Systematic review

## Abstract

Evidence-based allergology for the treatment of allergic rhinitis with allergen-specific immunotherapy (AIT) has been used in publications by the companies manufacturing AIT. The purpose of randomized controlled trials (RCTs) is to provide physicians, health authorities, patients, and their families with the best evidence upon which to base treatment decisions. However, some RCT results may do more harm than good because they serve the commercial interests of the companies producing and marketing AIT more than the interests of patients. Allergic rhinitis is a trivial disease that is not life-threatening and is easily controlled by drugs. In this paper, we analyze some of the more controversial points underlying the EBM supporting the use of AIT.

The paradox behind RCT-based practice is that AIT is based on the results of incorrectly interpreted RCTs. International scientific societies and drug regulatory bodies should analyze trials more carefully, considering potential conflicts of interest.

**Key points**

**Question:** How much is the evidence-based medicine (EBM) in allergology influenced by authors’ conflicts of interest?

**Findings:** In this review, we have analyzed the controversial field of allergen-specific immunotherapy (AIT), showing how the indications given by drug regulators and international scientific societies are influenced not only by economic conflicts of interest (COIs) but, above all, by academic ones, with repercussions for both patients and national health systems.

**Meaning:** Randomized controlled trials, when affected by obvious COIs, can cause EBM indications to be misleading. Greater clarity and transparency are needed in the interest of patients, avoiding the practice of discrediting authors who present different points of view, and trying instead to establish constructive scientific relationships.

## 1. Introduction

**Fig. 1:**
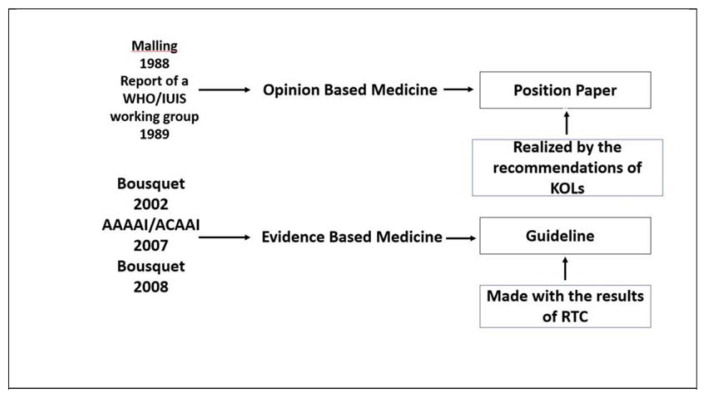
The shift from opinion-based medicine to evidence-based medicine. AAAAI: American Academy of Allergy, Asthma & Immunology; ACAAI: American College of Allergy, Asthma and Immunology; KOLs: Key Opinion Leaders; IUIS: International Union of Immunological Societies; RCT: randomized controlled trials; WHO: World Health Organization.

The evidence-based medicine (EBM) approach has influenced physician decision-making profoundly over the past 30 years [1]. As is well known, the founders of EBM, Cochrane, Feinstein and Sachett, shifted decision-making from subjective judgments (based on the experience and opinion of the senior physician) to a formal analysis of the evidence which, with Shekelle, Alan and Guyatt, led to the drafting of guidelines, written with different methodologies up to GRADE [2–4]. In other words, there was a shift from opinion-based medicine, i.e. position papers in which a treatment was recommended if physicians believed that patients could benefit from it, to EBM, i.e., guidelines in which a treatment was recommended based on the results of the evidence (Fig. 1) [5].

**Fig. 2:**
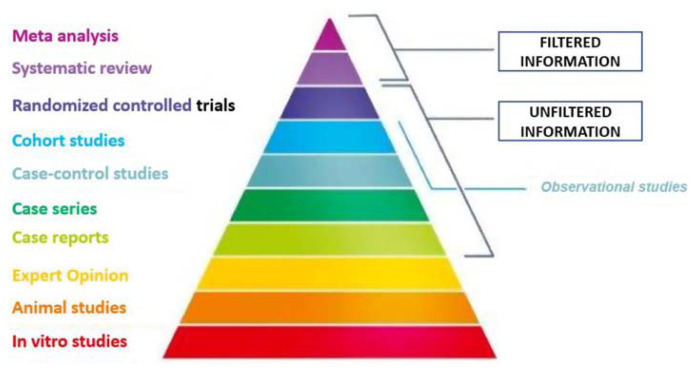
The pyramid of evidence.

But what is the evidence? Everybody knows the EBM pyramid, at the top of which is the systematic review (RS), with or without meta-analysis, of randomized controlled trials (RCTs) (Fig. 2).

The RS is a systematic analysis of RCTs which, in turn, are referred to as the gold standard for the “unbiased” evaluation of a treatment [6]. The concept of the impartiality of RCTs is due to the inclusion of at least one control group, which is compared with at least one intervention group which undergoes an experimental treatment. The control group may receive either a placebo (placebo-controlled study) or an already used “effective treatment” (positive control study). Another important feature of these studies is “randomization”: subjects are assigned to the treatment or control groups randomly. The randomization sequence can be obtained through several methodologies, but recently the use of software has consistently replaced other ones [7]. If both the physician/experimenter and the patient do not know which treatment is assigned, it is a double-blind placebo-controlled study; whereas, if it is only the patient who does not know the treatment, it is a single-blind placebo-controlled study. However, as pointed out by Ioannidis in one of his landmark publications [8], many of the results of RCTs evaluating a new treatment may not be entirely reliable because, as is well known, RCTs are often sponsored by the very companies that manufacture and market the drug being examined. This problem has been well highlighted with antidepressant drugs [9]. Ioannidis’s considerations point-out that, while the physician’s primary goal is to improve the patient’s health status, the pharmaceutical company’s is to increase financial profit for its shareholders [8]. in example of this is Vioox® with the ADVANCE study [10].

Thus, in an RCT, there can be an important conflict of interest (COI) in favor of the use of a therapy, which manifests itself with key opinion leaders (KOLs). The COI of KOLs, who are independent consultants to a pharmaceutical company, can be economic, which is easily identified because it is almost always declared, but also academic, which is rarely declared and is more difficult to identify. In many fields of medicine, the two COIs are often interconnected [11].

In allergology, the therapy for respiratory allergic diseases includes environmental preventive measures, medications, and allergen-specific immunotherapy (AIT) [12]. In this paper, we will deal with AIT, considering it with the EBM methodology.

The global AIT market in 2018 was estimated by GlobalData to be $901 million, with a projected increase to $1.1 billion in 2028 and with an average compound annual growth rate (CAGR) of 2.3%; thus, it is predicted be a very slow-growing market [13].

### 1.1. When, why, and how can a COI materialize?

Psychiatrist Christiaan Vinkers and his collaborators selected and analyzed publications that contained positive or negative words about therapies. The number of articles with positive words, in the title or abstract of the publication, increased from an average of 2.0% in 1974–80 to 17.5% in 2014, while articles with negative words increased from 1.3% to 2.4% over the same period [14].

Let us analyze when, why, and how a COI can arise for physicians who are the KOLs of a pharmaceutical company:

- When: if negative judgments are used in a publication for a treatment which, instead, the KOLs have validated with the company’s RCTs and RSs;
- Why: a negative judgment, if documented, calls into question what was previously published;
- How: first by attacking the author of the negative publication, and then by excluding him or her from the debate. To do this, KOLs minimize the importance of the study by highlighting points that KOLs consider weak, or by publishing the critique as correspondence, or by publishing an article in a journal that gives no opportunity to respond to criticism.

It is also important, when evaluating a study, to know the history of its peer reviews. Reviewers, in fact, are ‘Peer Reviewers’ chosen by the Editor, and they themselves may have not only an economic but also an academic COI for the work under evaluation, thus influencing the Editor’s decision for the outcome of a paper with their judgments [14]. The academic COI, in our opinion, is the most insidious, because it can block publication of a study that interferes with a strand of the peer reviewer’s research or because it highlights the topic’s lack of scientific importance, jeopardizing his/her position as Chief Researcher or his/her career progression [15,16].

In this way, a paper that criticizes diagnosis and therapy in a specialty branch, with data highlighting the scientific inconsistency of the claims of the KOLs of scientific societies, will be unlikely to be published by a journal of branch reference even if the observations are correct, noting rather the corporatist defense of specialized methods, such as *in vivo* diagnostics and AIT in allergology. In these cases, authors will have to seek space in a non-specialty journal. Thus, even if the non-specialty journal is extremely authoritative, this may result in a lack of the appropriate visibility of the paper for specialists in the field compared to the contrasting positions published in specialty-specific journals. Indeed, the specialist in the field practices according to the rules of the relevant scientific society; but, if these are questioned, the specialist’s own professional activity is questioned. This was the case with Holt’s article in 1967, published in the *New England Journal of Medicine* precisely because, in those years, its negative comments on allergy diagnostics and allergen-specific therapy were extremely provocative [17]. Francis Lowell, the President *pro tempore* of the American Academy of Allergy, Asthma & Immunology (AAAAI), hastened to write a very negative commentary on Holt’s article, which was, of course, published in the *Journal of Allergy and Clinical Immunology*, AAAAI’s flagship journal [18]. Holt had questioned *in vivo* allergy diagnostics, which at the time was the only diagnostic method practiced [17], along with the results of subcutaneous immunotherapy (SCIT), the only route of AIT administration practiced by allergists worldwide. In our opinion, it is important to remark that, while Holt’s article was published in *New England Journal of Medicine*, the critique was published in a journal with a specialty focus, the reference point of a major scientific society of allergology, and, therefore, widely read by specialists in the field.

## 2. Analysis of the EBM on AIT

In recent years, the importance of *in vivo* testing alone for diagnosing and prescribing AIT has been greatly downgraded [19], as has that of SCIT, the use of which has greatly diminished due to the use of sublingual immunotherapy (SLIT) [20]. SLIT can be administered in drops (SLIT-D) or tablets (SLIT-T); the former is a product designated as a Named Patient Product (NPP), as is SCIT.

### 2.1. Historical background

SLIT was approved by the FDA in 2014 but is not a new therapy, having been widely used in the United States for many years. The early literature includes many articles by a variety of authors [21–26]. As reported by Hansel [21], in 1928 Black was the first to identify the successful management of a pollen allergy by SLIT. Moreover, the American Academy of Otolaryngic Allergy offered a course called “Sublingual Therapy in Allergy” from 1963 to 1980. In that period, SLIT was available in two different forms: drops and rapidly dissolving tablets. These latter have only recently been introduced in Europe [27].

The use of SLIT in the United States decreased markedly and almost “vanished” (an exception is the American Academy of Environmental Medicine, where Max Samter offered courses on sublingual treatments for years) [28]. With the exception of a few publications by David Morris [29], no further articles about SLIT were published in the United States between 1970 and 1993, when Nelson et al. [30] performed an RCT concluding that SLIT with high-dose standardized cat extracts was no more effective than placebo in reducing symptoms or affecting immunological measures of cat sensitivity.

The prescription of SCIT and SLIT-D is specific and nominal for an individual patient, and is shipped directly to their home, with an exclusive company-patient relationship borrowed from the prescribing physician. Only a few extracts have been registered as “drugs” by the European Medicines Agency (EMA) and the Food and Drug Administration (FDA): Grazax®/Grastek®, sold in the EU by ALK and in the US by Merck, Sharp & Dohme, and Oralair® (Stallergenes/Greer) for respiratory allergy to grasses, and Accarizax®/Odactra® for respiratory allergy to house dust mites, sold in the EU by ALK and in the US by Merck, Sharp & Dohme. In Italy, Grazax® and Oralair® are dispensed by the national health system as SLIT-T, with a therapeutic plan and a spending cap [31,32].

SLIT has become the most prescribed route of administration in many EU countries which, however, still prefer SLIT-D over SLIT-T [33].

But, the question remains, whether what the regulatory agencies have approved has really been done right.

Many researchers are calling for more attention from regulatory agencies when evaluating the efficacy of a drug [34], and they may be right. Our misgivings are supported by the results of the RCT by Cox et al. [35], which is the only RCT performed in the US used by the FDA to authorize the marketing of SLIT-T (Oralair®) for grass rhinoconjunctivitis [36].

### 2.2. Criticalities present in Cox et al.’s RCT

#### 2.2.1. Authors

We will evaluate the study by Cox et al. [35] to highlight some critical issues that emerge from the careful reading of this research, but which were omitted by the SLIT KOLs [37,38].

Curiously, the authors of Cox’s RCT include three employees (Laurence Ambroisine, MSc, Michel Melac, MD, and Robert K. Zeldint, MD) of the company (Stallergenes SA, Antony, France) that sponsored the study. Only L. S. Cox, T. B. Casale, D. I. Bernstein, and P. S. Creticos report potential COIs, while the three Stallergenes employees declare no potential COIs. The role of each author in the publication is unknown because it is not stated.

#### 2.2.2. Methods

##### 2.2.2.1. Participants

The study enrolled men and women aged 18–65 years with documented grass pollen-related allergic rhinoconjunctivitis (ARC) for at least the 2 previous grass pollen seasons, a positive skin prick test (SPT) response to timothy grass (mean wheal diameter of 5 mm or greater than that elicited by the negative control; longest flare dimension, ≥10 mm), a retrospective Rhinoconjunctivitis Total Symptom Score (RTSS; scale, 0–18) of 12 or greater during the previous grass pollen season, and a Forced Expiratory Volume in the 1st second (FEV1) of 80% or greater of predicted value.

##### 2.2.2.2. Outcome

Participants were provided with a daily record card for recording the 6 individual Rhinoconjunctivitis Symptom Scores (RSSs: sneezing, runny nose, itchy nose, nasal congestion, itchy eyes, and watery eyes) and rescue medication use during the previous 24 hours. The diary cards were to be completed at the same time every evening, from approximately 3 weeks before the pollen season until its end, by using a 4-point descriptor scale for each symptom: 0, no symptoms; 1, mild symptoms; 2, moderate symptoms; and 3, severe symptoms. The daily RTSS was the sum of the 6 individual RSSs. The daily Rescue Medication Score (RMS) was derived as follows: 0, no rescue medication taken; 1, use of antihistamine (oral drops, eye drops, or both); 2, use of nasal corticosteroid; and 3, use of oral corticosteroid. If a study subject took 2 or more rescue medications on the same day, the highest score was used for the RMS.

Such a value, assuming an average Adjusted Symptom Score (AdSS) of 6 for the placebo group (as observed in previous grass pollen allergen studies), corresponds to a 20% relative mean difference, the threshold recommended by the World Allergy Organization (WAO) taskforce as clinically relevant for efficacy [37]. Assuming a screening failure rate of 20% and a dropout rate of 10%, the authors planned to screen approximately 550 subjects and randomize 424 subjects.

##### 2.2.2.3. Statistical analysis

The primary efficacy end point [daily combined score (CS) during the pollen period while receiving treatment] was analyzed for the full analysis set by using a repeated measures analysis of covariance model, including treatment group and valid days as main effects, and patient as a random effect. The daily RTSS, RMS, AdSS, and RSSs were analyzed as per the primary efficacy criterion. We will only consider the RTSS because it is a parameter that was also used to recruit the participants (retrospective RTSS) of the study, in order to ensure the equivalence of rhinitis severity in the two treatment groups.

##### 2.2.2.4. Results

Four hundred seventy-three participants were randomized for active treatment (n = 233) or placebo (n = 240). Because 35 study subjects did not have at least one CS during the pollen period while receiving treatment, the full analysis set (FAS) consisted of 438 participants: 210 in the 300IR group and 228 in the placebo group. The mean duration of ARC was 22.9 years (SD, 12.8 years).

**Fig. 3:**
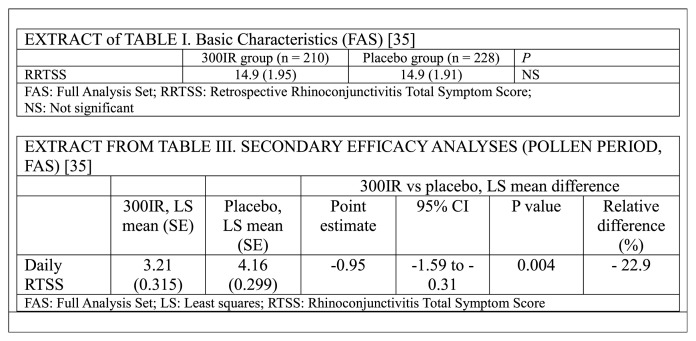
Extracts of table I and table III of the original data of Cox et al. [35].

Fig. 3 shows an extract of the original retrospective RTSS and RTSS data from the original article by Cox et al. [35] Specifically, we report only the data of the retrospective RTSS (Table I) and the RTSS (Table III).

**Fig. 4:**
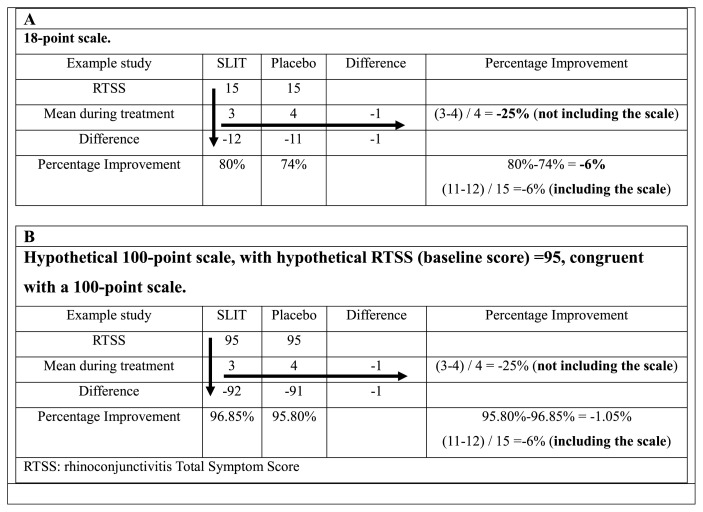
A and B mathematical demonstration of the fallacy of the calculation performed by Cox and proposed by World Allergy Organization [39].

More specifically, we will focus our analysis on the most critical methodological problem of the RCTs on SLIT-T: the metric used by the authors to evaluate the clinical benefit. This metric is mathematically incorrect because it calculates the percentage difference between SLIT and placebo, not considering the range of the symptom score (SS) scale and enormously magnifying the difference between the groups. Using this metric, a 1-point difference between the two treatments would have the same percentage difference on an 18-point scale (the most used SS scale) as it would on a 100-point scale or any other scale, and this is mathematically unacceptable (Fig. 4A and B).

In Fig. 4 A and B, we show the calculation used in RCTs (horizontal arrow), which considers only the mean SS at the end of treatment, ignoring the scaling interval. The use of the latter is, in fact, critical in assessing the efficacy of SLIT-T vs. Placebo. Including the scale in the calculation significatively reduces the percentage of improvement, although the difference between the 2 groups remains the same. Alternatively, the difference between the groups can be calculated as follows: SSt SLIT (t = during treatment) − SSt Placebo (t = during treatment)/SSu (u = SS without treatment), thus the baseline value of the SLIT group and the placebo group, which are equal at the time of randomization (Baseline characteristics [35]). This calculation allows us to use the range of the symptom scale to assess clinical improvement, unlike the method used (SLIT SSt − Placebo SSt/Placebo SSt [t = during treatment]), which is incorrect in not considering the range of the symptom scale used and, thus, overestimating the treatment effect. The correct metric, which must consider the range of the scale, has been given by the WAO [37] and is based on the comparison of pre-treatment and post-treatment SS from the intervention and placebo groups. Using this metric, only a small difference between SLIT and placebo is demonstrated, far below the FDA (15%) and WAO (20%) efficacy thresholds [37,38]. The baseline value in the case of SLIT RCTs is the RTSS score (previous year), which is used by the investigators as an inclusion criterion. In other words, the RTSS score is taken in RCTs as the SS that patients would have in the absence of treatment, and it corresponds to the clinical criterion (severity of rhinitis) for inclusion. Certainly, the RTSS could be inaccurate, but it should be similar to the SS of the treatment season, especially if the pollen counts of the two consecutive seasons are similar, and this possible inaccuracy should not affect the results.

In a meta-analysis, our group [39] also reported the difference between SLIT and placebo not only in terms of standardized mean difference (SMD), but also in terms of mean difference (MD), that is, the difference in SS points between SLIT and placebo. We showed that this difference is −0.83 SS points (95% CI, −1.03 to −0.63). In 2014, Devillier et al. [40] published that the minimally clinically important difference, defined as the smallest improvement considered beneficial by a patient, is between 1.1 and 1.3 SS points in patients with ARC from grass pollen. Therefore, the difference of −0.83 SS points reported in the previously published meta-analysis [39] is less than the minimal clinically important difference estimated by Devillier [40].

In conclusion, analyses based on Devillier’s minimum important difference, which sets the efficacy threshold at 1.1 SS points, confirm the conclusions of our meta-analysis [39] which estimated a small benefit from SLIT-T, lower than the FDA’s 15% or WAO’s 20% difference thresholds, and that the flawed metric used in the RCT of Cox and the other RCTs with grass SLIT-T (*see RCTs reported in reference* [39]) significantly overestimated the modest, and perhaps clinically unimportant, treatment benefit.

Another observation on the fatuity of allergy guidelines can be made again by analyzing Cox’s study which, unlike all other RCTs with SLIT-T, did not consider the presence of serum IgE concentration ≥0.7 kilo allergy unit per liter (KUA/L) among the inclusion criteria [37,41]. Cox et al. [35], in discussion, stated that they were consistent with the behavior of US allergists, and perhaps many non-US allergists as well, who in their daily practice base allergological diagnoses and prescriptions of AIT on SPT alone [35]. The authors report, however, that in 11% of the subjects enrolled in their study, the serum IgE specific for grasses was <1 KUA/L, that is, they were not allergic to grasses. One wonders, again, how these patients could be considered in the study and whether (and how much) their inclusion and then exclusion vitiated the reported results, even though they were not considered in the statistical analysis [35].

## 3. Discussion

### 3.1. Who controls the controllers?

In the meta-analysis of our group [39], the results obtained demonstrated only a small benefit of SLIT-T for seasonal ARC to grass pollen for symptoms and symptomatic drug use (antihistamines and nasal corticosteroids), and the conclusions were congruent with these findings: considering the low magnitude of benefit, convenience and ease of administration do not seem to be sufficient reasons for choosing SLIT-T.

These conclusions have sparked reactions by some Stallergenes KOLs (*see below*). Devellier [42], in the ‘Translating Best Evidence into Best Care’ section of the *Journal of Pediatrics*, after several criticisms of the meta-analysis [39], concluded, “Although the methodology of their meta-analysis was rigorous, their interpretation of the results is questionable.” As soon as we read this comment, we wrote to the Editor *pro tempore* of the journal, Professor WF Balistrieri, who did not want to publish our response to Devillier nor our request to clarify the sentence “their interpretation of the results is questionable,” with which he had concluded his letter. Professor Balistrieri sent me an e-mail in which he wrote that “our reply was not a priority for the Journal.” A personal e-mail was, in addition, sent to Devellier (p.devillier@hopital-foch.org), but we never received a reply.

Finally, we feel it is important to point out that the Stallergenes KOLs themselves published their critique as a correspondence, not in *JAMA Internal Medicine* where the original article had been published, but in another journal, and they concluded with the following sentence: “In conclusion, Di Bona et al. performed a rigorous meta-analysis but over-interpreted the results while losing sight of other important parameters.” [43] Again, they were asked to contextualize what the above sentence referred to, but the clarifying reply never came.

In 2017, Passalacqua, Incorvaia and Ridolo, referring to our meta-analysis [39], wrote: “These conclusions claim to wipe out all the long process leading to the acknowledgement of the quality and to registration of grass pollen tablets, but are substantially unfounded because the meta-analysis is significantly flawed, as highlighted by Cox et al.” [43,44]. From a simple PubMed search as of October 20 2022, we found that: Passalacqua, using the search [(Passalacqua G [Author]) AND (allergen immunotherapy)], has as many as 209 publications on AIT, placing him as the first researcher in the world in terms of number of publications on this topic; Incorvaia, a scientific advisor for Stallergenes Italy, using for research [(Incorvaia C [Author]) AND (allergen immunotherapy)], has 151 publications on AIT; Ridolo, using the search [(Ridolo E [Author]) AND (allergen immunotherapy)], has 54 publications on AIT. We also find it interesting to note that Passalacqua and Ridolo have never claimed to have any COIs. The article and this bibliometric check suggest a COI which is not economic, but academic because, in our opinion, it is difficult to explain such a high number of publications for a low-efficacy therapy.

## 4. Conclusions

Many KOLs have created academic space for themselves by exploiting EBM for diseases with high epidemiological impact but low clinical relevance, while proper drug therapy optimally controls the symptoms of seasonal ARC [45].

As internists, we would like to conclude by paraphrasing a 1904 aphorism by William Osler, which is still very relevant despite the 118 years that have passed: “There are two kinds of researchers, those who do research using their brains, and those who do research using only their mouths.”

## Abbreviations

AAAAI: American Academy of Allergy, Asthma & Immunology
ACAAI: American College of Allergy, Asthma and Immunology
AdSS: Adjusted Symptom Score
ARC: Allergic rhinoconjunctivitis
AIT: Allergen-specific immunotherapy
COI: Conflict of interest
CAGR: Average compound annual growth rate
CS: Combined score
EBM: Evidence-based Medicine
EMA: European Medicines Agency
FAS: Full Analysis Set
FDA: Food and Drug Administration
FEV1: Forced Expiratory Volume in the 1st second
GRADE: Grading of Recommendations, Assessment, Development and Evaluation
KOL: Key Opinion Leaders
KU_A_/L: Kilo allergy unit per liter
IR: Index of reactivity
LS: Least squares
MA: Meta analysis
MS: Medication score
RCT: Randomized controlled trials
RMS: Rescue Medication Score
RTSS: Rhinoconjunctivitis Total Symptom Score
SCIT: Subcutaneous immunotherapy
SLIT-D: Sublingual immunotherapy Drop
SLIT-T: Sublingual immunotherapy Tablet
SPT: Skin Prick Test
SS: Symptom score
SR: Systematic review
WAO: World Allergy Organization
WHO: World Health Organization
